# Sub-half-cycle field transients from shock-wave-assisted soliton self-compression

**DOI:** 10.1038/s41598-020-67134-y

**Published:** 2020-07-23

**Authors:** A. A. Voronin, A. M. Zheltikov

**Affiliations:** 1 Physics Department, International Laser Center, M.V. Lomonosov Moscow State University, Moscow, 119992 Russia; 2 Department of Physics and Astronomy, Texas A&M University, College Station, TX 77843 USA; 3 Russian Quantum Center, Skolkovo, Moscow Region 143025 Russia; 4 Kazan Quantum Center, A.N. Tupolev Kazan National Research Technical University, Kazan, 420126 Russia

**Keywords:** Ultrafast photonics, Ultrafast photonics

## Abstract

We identify an unusual regime of ultrafast nonlinear dynamics in which an optical shock wave couples to soliton self-compression, steepening the tail of the pulse, thus yielding self-compressing soliton transients as short as the field sub-half-cycle. We demonstrate that this extreme pulse self-compression scenario can help generate sub-half-cycle mid-infrared pulses in a broad class of anomalously dispersive optical waveguide systems.

## Introduction

Extremely short, subcycle electromagnetic field waveforms are rapidly emerging as powerful tools for ultrafast optics and photonic technologies^1–3^, enabling an unprecedented, subfemtosecond time resolution in laser spectroscopy^4,5^ and an ultimate, subcycle precision in lightwave sculpting^6,7^. Subcycle field waveforms are not unusual in terahertz technologies^8^, where such waveforms are generated as a result of optical rectification^9,10^, providing means for time-domain spectroscopy^11^, terahertz sensing^12^, and sub-quarter-cycle engineering^13^. Terahertz field cycles are, clearly, too long to be relevant as probes for ultrafast dynamics in molecules, let alone the attosecond dynamics of electron wave packets and electron excitations in atoms and solids. Yet, the significance of the early work on subcycle terahertz field transients^8,9,14^ is hard to overestimate – we owe it much of our understanding of fundamental properties of subcycle pulses and the universal tendencies in their unusual propagation dynamics.

Optical methods of subcycle pulse generation rely on coherent field waveform synthesis^2^ operating with high-order harmonics^15^, multiple Raman sidebands^16–19^, frequency-shifted supercontinua from hollow-core fibers^1,6^, and cascaded parametric amplification^20^. As a promising alternative, anomalous-dispersion-assisted multioctave supercontinuum generation in solid materials^21^ and guided-wave soliton self-compression (SSC)^22,23^ can help create efficient sources of subcycle pulses covering a broad range of frequencies and peak powers.

Here, we identify an unusual regime of ultrafast nonlinear dynamics in which an optical shock wave couples to soliton self-compression, steepening the tail of the pulse, thus yielding self-compressing soliton transients as short as the field sub-half-cycle. Optical shock waves are inevitable when the pulse duration of a field waveform approaches the field cycle^24^. As their archetypical signature, optical shock waves tend to steepen the tail of the pulse, blue-shifting its spectrum^25^. In special regimes of three-dimensional free-beam propagation, optical shock waves have been shown to force field waveforms to self-compress to subcycle pulse widths^26^. Ordinarily, however, when building up via waveguide short-pulse evolution, optical shock waves do not lead to pulse shortening as a whole, giving rise to pulses with sharper trailing edges and asymmetric supercontinua.

Still, our analysis presented in this paper shows that, when coupled to soliton self-compression an optical shock facilitates the generation of extraordinarily short field waveform transients, giving rise to soliton transients shorter than the field half-cycle. As we demonstrate below in this paper, this scenario of extreme pulse self-compression can help generate sub-half-cycle pulses in the mid-infrared range and can be implemented in a class of fibers with generic dispersion properties typical of antiresonance-guiding hollow-core photonic-crystal fibers (PCFs).

### Broadband anomalous dispersion

SSC-based generation of subcycle field waveforms is only possible in a waveguide that can support anomalously dispersive low-loss guiding within more than an octave around the central frequency of the driver. Kagome-cladding^27,28^ and single-ring antiresonance-guiding (AR)^29,30^ hollow-core (HC) PCFs can support such propagation regimes for both near-IR^23,31–33^ and mid-IR laser pulses^34^.

**Figure 1:**
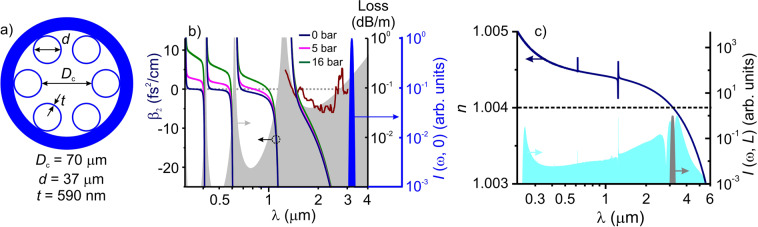
(**a**) Single-ring hollow-core photonic-crystal fiber for soliton self-compression in the mid-infrared. (**b**) Group velocity dispersion of the single-ring hollow PCF filled with argon at the pressure *p* = 0 bar (blue line), 5 bar (pink line), and 16 bar (green line). The fiber loss spectrum is shown by grey shading (calculations) and maroon line (experiment^38^). Also shown is the spectrum of the mid-IR driver pulse (blue shading). (**c**) The effective refractive index as a function of the wavelength for the fundamental mode of the antiresonance-guiding hollow PCFs as shown in (**a**). The fiber is filled with argon at a pressure of 16 atm. The dotted line shows the median value *n*_0_ of the refractive index. Also shown the spectrum of a subcycle pulse generated as a result of soliton self-compression in this fiber (blue shading) and the spectrum of the input mid-IR pulse (grey shading).

Here, we show that suitable dispersion and transmission are found in the class of hollow-core PCF with a single-ring AR coating. Such fibers have been earlier shown to enable the generation of multioctave supercontinua in the near- and mid-IR^31–34^. As our specific choice of the parameter space, fine-tuned toward achieving the targeted fiber dispersion and transmission properties, we take an AR hollow PCF with a core diameter *D*_c_ ≈ 70 µm and a single-ring AR cladding of six identical silica rings with a diameter *d* ≈ 37 µm each, bounded by an outer wall of thickness *t* ≈ 0.59 µm (Fig. 1a). Figure 1b displays the loss and group-velocity dispersion (GVD) of this fiber calculated^35^ as functions of the radiation wavelength. A PCF with such a structure supports low-loss, anomalous-GVD guided modes (the solid line and grey shading in Fig. 1b) within the entire bandwidth covered by an output of mid-IR optical parametric amplifiers (OPAs) that have recently emerged as attractive sources for ultrafast strong-field nonlinear-optical studies in the mid-IR^36,37^. The broadband GVD anomaly provided by this fiber allows the entire spectrum of the sub-200-fs output of such OPA sources to be coupled into a soliton pulse inside the fiber. Calculations for the loss of this fiber agree well (cf. the grey shading and the maroon line in Fig. 1b) with the available experimental data^38^, verifying the predictive power of the model.

### Soliton self-compression to subcycle pulse widths: physical model

#### General framework

Aiming for a formalism that would be applicable to extremely short, subcycle field waveforms, we adopt, as our starting point, the following general definition of the complex electric field:

1 $$A(\eta ,z)={\int }_{0}^{\infty }E(\omega ,z)\exp (-i\omega \eta )d\omega ,$$

where *E*(*ω, z*) = ${\int }_{-\infty }^{\infty }E$(*η, z*)exp(*iωη*)*dη* is the spectrum of the real-valued electric field *E*(*η, z*) and *z* is the propagation coordinate.

The real-valued electric field is recovered from its complex counterpart via

2 $$E(\eta ,z)=A(\eta ,z)+A\ast (\eta ,z).$$

For an ultrashort pulse with an electric field component as defined by Eq. (2), the real-valued magnetic field component in a nonmagnetic medium with a frequency-dependent complex dielectric function *ε*(*ω*) is written as

3 $$H(\eta ,z)=c{\varepsilon }_{0}{\int }_{-\infty }^{\infty }{[\varepsilon (\omega )]}^{1/2}E(\omega ,z)\exp (\mbox{--}i\omega \eta )d\omega ,$$

where [*ε*(*ω*)]^1/2^ = *n*(*ω*) + *iκ*(*ω*) is the dielectric function, *n*(*ω*) and *κ*(*ω*) are the refractive index and loss, *c* is the speed of light in vacuum, and *ε*_0_ is the vacuum permittivity.

Equations (1–3) are well-suited for the purposes of our study as they define optical fields without resorting to a notion of the central frequency, which tends to become awkward when applied to subcycle pulses.

The intensity of an ultrashort electromagnetic pulse is defined, in accordance with a standard self-consistent prescription, as the time average of the *z*-component of the Poynting vector,

4 $$I(\eta ,z)= < S(\eta ,z) > $$

When extended to subcycle electromagnetic pulses, however, this definition of field intensity may encounter serious difficulties. Indeed, with electric and magnetic fields in an ultrashort electromagnetic pulse defined by Eqs. (1–3), Eq. (4) leads to

5 $$S(\eta ,z)=c{\varepsilon }_{0}E(\eta ,z){\int }_{-\infty }^{\infty }{[\varepsilon (\omega )]}^{1/2}E(\omega ,z)\exp (\mbox{--}i\omega \eta )d\omega .$$

It is straightforward to see from Eq. (5) that, for a broadband field waveform propagating in a medium with a strong dispersion of *ε*(*ω*), *<S*(*η, z*)> does not necessarily provide a good measure of [*E*(*η, z*)]^2^, as would be the case of many-cycle pulses. Instead, when the spectrum of a pulse becomes so broad that the dispersion of *ε*(*ω*) is no longer negligible, the integral in Eq. (5) does not reduce to a simple Fourier transform of *E*(*ω*, *z*).

In the context of this work, that is, subcycle pulse generation in optical fibers, this problem is, perhaps, best addressed by examining a typical behavior of *n*(*ω*) and *κ*(*ω*) found in a broad class of hollow-core PCFs used in ultrafast photonic technologies^23,29–34,38^, including antiresonance-guiding hollow PCFs as shown in Fig. 1a. In Fig. 1c, we plot the refractive index *n* as a function of the wavelength found by solving the dispersion relation for the fundamental mode of this fiber when filled with argon at a pressure of 16 atm. Fibers of this type have been shown^34^ to enable efficient soliton self-compression of mid-infrared laser pulses. The related wavelength dependence of the fiber loss, as dictated by *κ*(*ω*), is presented in Fig. 1b. The most important conclusion to be drawn from the *n*(*ω*) profile in Fig. 1c is that, across the range of wavelengths from 0.2 to 6 µm, i.e., across a bandwidth that is sufficient to support a subcycle laser pulse, the refractive index changes by as little as |*δn* | ≈ 0.001*n*_0_, *n*_0_ being the median value of *n*(*ω*) within this wavelength range, as shown by the dotted line in Fig. 1c. Although the behavior of *n*(*ω*) depends on a specific fiber design and fiber parameters, within the fiber transmission range, |*δn* | is typically way below 1% of *n*_0_ for a broad class of anti-resonance- and inhibited-coupling-guiding hollow-core PCFs used in short-pulse experiments^23,29–34,38^.

Since *δn*/*n*_0_ ≪ 1 in the class of *n*(*ω*) profiles pertinent to this study, we set *n*(*ω*) ≈ *n*_0_ in Eq. (5). Averaging the resulting approximate expression for *S*(*η, z*) over time and discarding small corrections stemming from fast-oscillating terms, we find, by combining Eqs. (4) and (5)

6 $$I(\eta ,z)= < S(\eta ,z) > \approx (2{n}_{0}{e}_{0}c)|A(\eta ,z){|}^{2}.$$

Equation (6) recovers a comfortable relation between the field intensity and the amplitude of the complex field, commonly used in the analysis of many-cycle laser pulses. As we will show below, approximation of Eq. (6) does not give rise to any significant error in a definition of field intensity relative to Eq. (4).

### Evolution equation

Ultrafast dynamics of ultrashort laser pulses is analyzed in this study by solving a nonlinear evolution equation for the complex electric field as defined by Eq. (1). With ultrafast-ionization and harmonic-generation effects included^25,39–42^, we write this equation as

7 $$\begin{array}{c}\frac{\partial }{\partial z}A(\omega ,z)=i\hat{D}(\omega )A(\omega ,z)-\alpha (\omega )A(\omega ,z)\\ +i\frac{\omega \Theta (\omega )}{2cn(\omega )}\hat{F}\{3{\chi }^{(3)}{|A(\eta ,z)|}^{2}A(\eta ,z)+3{\chi }^{(3)}{|A(\eta ,z)|}^{2}{A}^{\ast }(\eta ,z)+{\chi }^{(3)}{A}^{3}(\eta ,z)\}\\ -\Theta (\omega )\hat{F}\left\{\frac{{U}_{i}W(I(\eta ,z))({\rho }_{0}-\rho (\eta ,z))}{2I(\eta ,z)}A(\eta ,z)\right\}\\ -\left(\frac{i{e}^{2}\omega }{2cn(\omega ){m}_{e}{\varepsilon }_{0}({\omega }^{2}+{\tau }_{c}^{-2})}+\frac{\sigma (\omega )}{2}\right)\Theta (\omega )\hat{F}[\rho (\eta ,z)A(\eta ,z)]\},\end{array}$$

Here, $\hat{F}$ is the Fourier transform operator, *A*(*ω*, *z*) = $\hat{F}$[*A*(*η*, *z*)], $\hat{D}$ = *β*(*ω*) − *ω*/*u* is the dispersion operator, *β*(*ω*) is the propagation constant, *η* = *t* − *z*/*u*, *χ*^(3)^ is the third-order nonlinear-optical susceptibility, *α*(*ω*) is the linear loss due to the mode leakage, Θ(*ω*) is the Heaviside step function, *ρ*(*η*, *z*) is the electron density, *W* is the photoionization rate, *U*_*i*_ = *U*_0_ + *U*_osc_, *U*_0_ is the ionization potential, *U*_osc_ is the energy of field-induced electron quiver motion, *m*_e_ and *e* are the electron mass and charge, *ρ*_0_ is the initial density of neutral species, and *σ*(*ω*) is the inverse bremsstrahlung cross section.

Equation (7) is solved jointly with the equation for the dynamics of the electron density,

8 $$\partial \rho /\partial \eta =W(I)+\sigma {U}_{i}^{-1}\rho I.$$

The rate of photoionization *W* in our model is calculated using the Popov–Perelomov–Terentyev model^43,44^. The cross section of inverse bremsstrahlung *σ* is calculated in the approximation of the Drude model, *σ*(*ω*) = *e*^2^*τ*_c_[*m*_e_ε_0_*n*(*ω*)*c*(1 + *ω*^2^*τ*_c_^2^)]^–1^, with *τ*_c_ being the collision time.

The initial temporal envelope and the input spectrum of the laser field are taken in the form of the temporal envelope and the spectrum of a typical short-pulse output of a multicascade mid-IR OPA^36,37^ with a central wavelength *λ*_0_ = 3.2 µm, pulse duration *τ*_0_ = 175 fs, and a spectrum as shown by blue shading in Fig. 1b. Field-intensity calculations are performed with a Bessel transverse beam profile, *f*(*r*) = *J*_0_(2.405*r*/*r*_c_), where *J*_0_(*x*) is the zeroth-order Bessel function, *r* is the radial coordinate, and *r*_c_ = *D*_c_/2 is the fiber core radius. For pulse compression in a hollow PCF filled with argon, the Kerr-effect nonlinear refractive index is *n*_2_ ≈ 1.35 × 10^−19^(*p*/*p*_a_) cm^2^/W, *p*_a_ is the atmospheric pressure, and the cubic susceptibility responsible for third-harmonic generation, and *χ*^(3)^ ≈ 3.2 ∙ 10^–22^(*p*/*p*_a_) cm^2^/V^2^. The ionization potential of argon is *U*_0_ ≈ 15.76 eV and the Drude-model collision time is *τ*_с_ ≈ 190(*p*_a_ /*p*) fs.

Although a hollow-core antiresonance-guiding PCF with the above-specified parameters is nominally multimode, regimes in which only one isolated guided mode is excited via a suitable beam coupling has been demonstrated in numerous experiments (see, e.g., refs. ^23,29–34^). For high-power laser pulses, however, the spatial self-action of the laser field due to the Kerr nonlinearity tends to couple waveguide modes, giving rise to energy transfer to higher order guided modes, as well as to leaky and tunneling modes. The critical power for such self-action phenomena is given by *P*_cr_ = *Cλ*^2^/(4*πn*_0_*n*_2_), where the constant *C* is independent of material parameters and is determined by the specific beam profile and boundary conditions. With generic boundary conditions for the field in a cylindrical hollow fiber, we have *C* ≈ 1.9^45,46^. For typical parameters of our calculations (*n*_2_ ≈ 1.35 ∙ 10^−19^ cm^2^/W for argon at *p* = 1 atm and *λ* = 3.2 µm), we then find *P*_cr_ ≈ 120 GW. Even higher values of *P*_cr_ are predicted by models where the constant *C* is defined as *C* = (${u}_{2}^{2}$ − ${u}_{1}^{2}$)/2, *u*_1_ and *u*_2_ being the eigenvalues of the lowest order waveguide modes^45–47^. With *u*_1_ ≈ 2.405 and *u*_2_ ≈ 5.52 for the LP_01_ and LP_02_ modes of a cylindrical waveguide, this model dictates *P*_cr_ ≈ 750 GW. Here, aiming at finding the lower-bound estimate for *P*_cr_, we take, following refs. ^45–47^, *C* ≈ 1.9, leading to *P*_cr_ ≈ 120 GW. The maximum peak power of laser pulses in our simulations (*W*_0_ = 50 µJ, *τ*_0_ = 175 fs) is *P* ≈ 0.3 GW, corresponding to *P*/*P*_cr_ ≈ 0.0025 ≪ 1.

We can appreciate now how important the *λ*^2^ scaling of *P*_cr_ is for the self-focusing-free transmission of high-peak-power mid-infrared pulses through a hollow fiber. Indeed, had our calculations been performed for the standard wavelength of Ti; sapphire laser radiation, *λ* = 0.8 µm, the critical peak power would have been more than an order of magnitude lower, *P*_cr_ ≈ 7.5 GW. In a recent experiment^34^, a single-ring hollow PCF was employed to demonstrate single-mode soliton compression of 3.25-µm laser pulses to a 1.35-cycle pulse width. In another experiment^23^, a kagome-cladding hollow PCF was used to implement soliton pulse compression to a nearly single-cycle pulse width at *λ* ≈ 1.8 µm. The maximum peak power *P* ≈ 0.3 GW was achieved in this experiment for the compressed soliton output, with the critical power of self-focusing being *P*_cr_ ≈ 2 GW. Even though the *P*/*P*_cr_ ratio in this experiment was ≈60 times higher than *P*/*P*_cr_ in our calculations, the compressed soliton output was generated in a single-mode regime without any noticeable energy transfer to higher order modes.

### Optical shock and sub-half-cycle soliton transients

**Figure 2:**
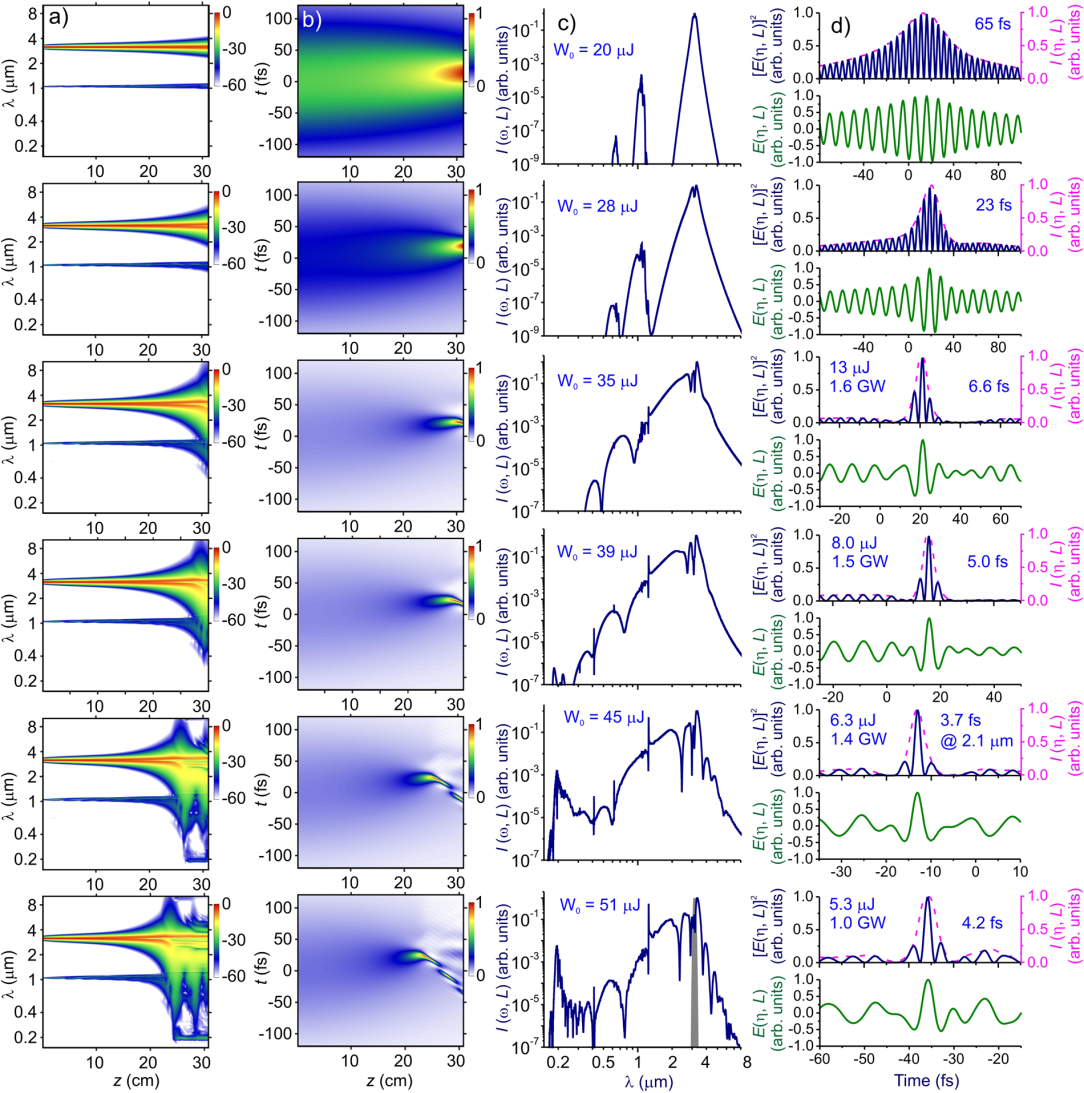
Spectral and temporal transformation of a mid-IR pulse in a single-ring hollow PCF filled with argon at *p* = 5.0 bar: (**a**) spectral intensity *I*(*ω, z*) = (2*n*_0_*ε*_0_*c*)|*A*(*ω, z*)|^2^, (**b**) time-domain field intensity *I*(*η, z*), (c) *I*(*ω, L*), and (d) *E*(*η, L*) (green solid line), [*E*(*η, L*]^2^ (blue solid line), and *I*(*η, L*) (red dashed line). The input energy of the driver pulse is as shown in the panels. The fiber length is *L* = 31 cm. The input spectrum of the driver is shown by grey shading.

**Figure 3:**
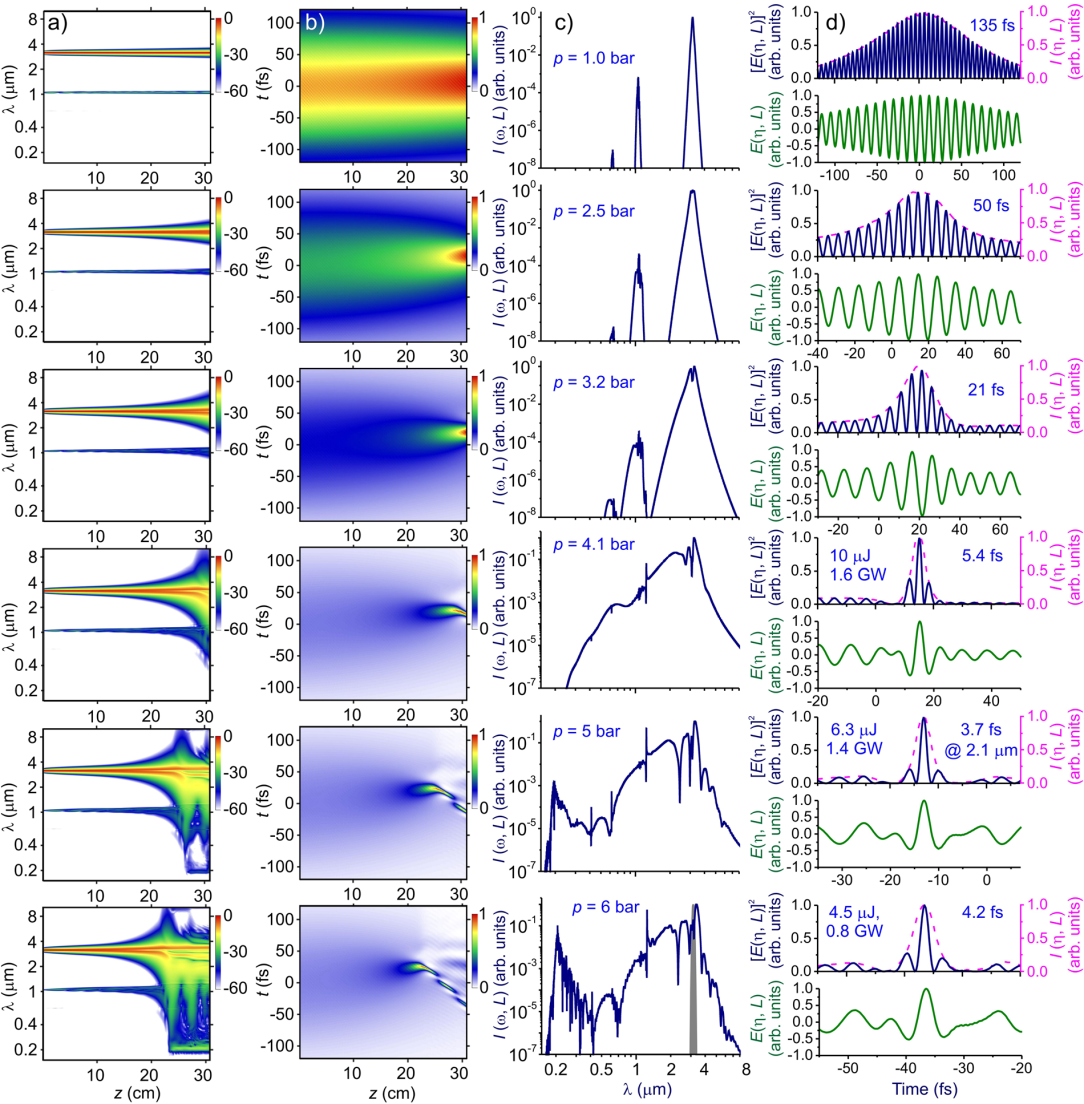
Spectral and temporal transformation of a mid-IR pulse in a single-ring hollow: (**a**) spectral intensity *I*(*ω, z*), (**b**) time-domain field intensity *I*(*η, z*), (**c**) *I*(*ω, L*), and (**d**) *E*(*η, L*) (green solid line), [*E*(*η, L*)]^2^ (blue solid line), and *I*(*η, L*) (red dashed line). The argon pressure is as shown in the panels. The input energy of the driver pulse is *W*_0_ = 45 μJ. The fiber length is *L* = 31 cm. The input spectrum of the driver is shown by grey shading. Temporal waveforms are shown with a dashed line.

In Fig. 2, we present the spectral and temporal evolution of ultrashort mid-IR pulses in a fiber with the above-specified parameters. For low levels of the input driver energy *W*_0_, the spectra of laser pulses exhibit almost symmetric broadening (Fig. 2a,c), indicating that, in this range of laser intensities, self-phase modulation provides a dominant mechanism of spectral broadening. At higher level of laser intensities and/or higher gas pressures, however, the spectral–temporal transformation of laser pulses becomes more complicated, as their spectra become dressed with optical harmonics (Figs. 2, 3), while the shock waves and ultrafast photoionization tend to show up, giving rise to a strong spectral blue shifting (Figs. 2a,c, 3a,c). That the gas-pressure scans of the output spectra remain similar to their input-driver-energy scans within a broad range of *W*_0_ and *p* indicates that the *pI*_0_ product (*I*_0_ being the input driver intensity) remains meaningful as a valid scaling parameter^48^ within a broad range of output bandwidths and pulse widths.

As a universal tendency of spectral transformation of the driver seen in Fig. 2a, the initial stage of a slow, gradual spectral broadening of the driver pulse (within the first 15–20 cm in Fig. 2a) is followed, in the case of high *W*_0_ and *p*, by a stage within which the driver bandwidth tends to build up in a dramatic, almost explosion-like manner, gaining more than an octave within just a few centimeters (20 < *z* < 26 cm in Fig. 2b at *W*_0_ = 45 μJ). Analysis of the driver dynamics in the time domain shows (Fig. 2b,d) that, as a part of this explosion-like supercontinuum buildup, the driver pulse undergoes self-compression to extraordinarily short waveform transients, with its pulse width becoming as short as the field sub-half-cycle (2.1 fs at the central wavelength of 2.1 μm) at the point of maximum pulse compression.

To gain insights into this pulse self-compression scenario, it is instructive to consider the dynamics of ideal optical solitons as an ultimate reference. To this end, we solve the NSE, that is, Eq. (1) with all the non-NSE terms disabled, for the same initial conditions and the same parameters of dispersion and nonlinearity. The dynamics of ideal NSE solitons can be understood in terms of the soliton number *N* = (*l*_d_/*l*_nl_)^1/2^, where ${l}_{d}={\tau }^{2}/|{\beta }_{2}|$, *l*_d_ is the dispersion length, *l*_nl_ = *λ*(2*πn*_2_*I*)^−1^ is the nonlinear length, *τ* is the pulse width, *β*_2_ is the group-velocity dispersion, and *λ* is the radiation wavelength.

**Figure 4:**
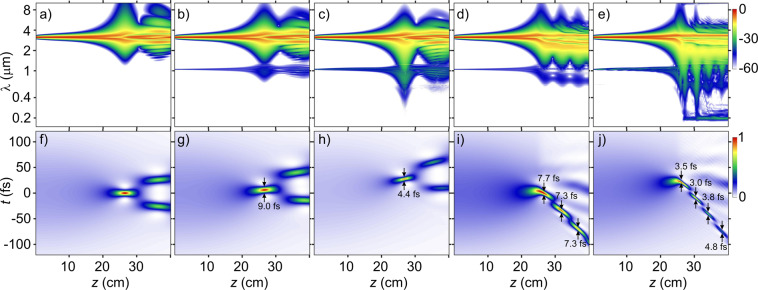
The maps of *I*(*ω, z*) (**a–e**) and *I*(*η, z*) (**f–j**) for a mid-IR pulse with *W*_0_ = 45 µJ in a single-ring hollow PCF filled with argon at *p* ≈ 5.0 bar simulated by solving (**a**,**f**) the NSE, (**b**,**g**) the GNSE without the ionization and self-steepening terms, (**c**,**h**) the GNSE without all the ionization terms, (**d**,**i**) the GNSE without the self-steepening term, and (**e**,**j**) the full GNSE.

**Figure 5:**
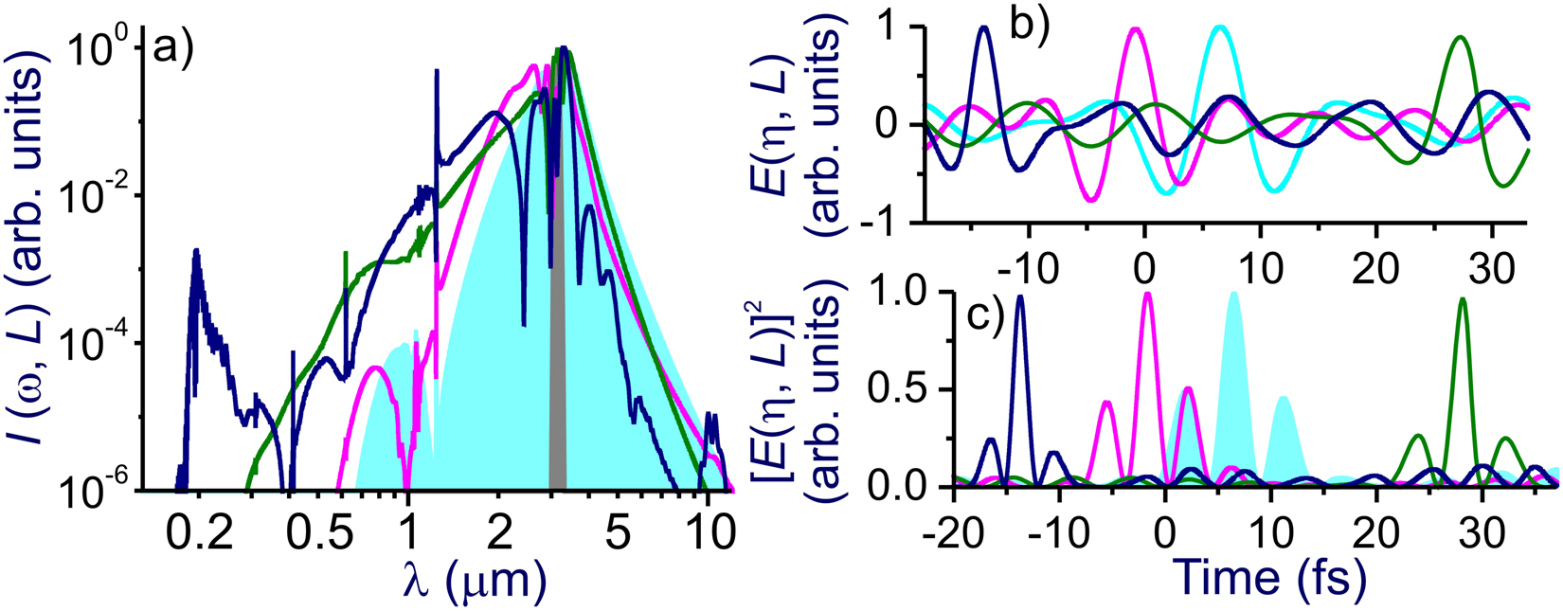
The spectral intensity *I*(*ω, L*) (**a**), the field *E*(*ω, L*) (**b**), and [*E*(*ω, L*)]^2^ (**c**) at the output of a single-ring hollow PCF simulated by solving the full GNSE [Eq. (1)] (blue line), the GNSE without all the ionization terms (green line), the GNSE without the self-steepening term (red line), and the GNSE without the ionization and self-steepening terms (cyan shading and line). The fiber is filled with argon at *p* ≈ 5 bar. The fiber length is *L* = 31 cm (blue) and 27 cm (green, red, and cyan). The input driver energy is *W*_0_ ≈ 45 µJ. The input spectrum of the driver is shown by grey shading.

The NSE soliton exhibits a signature breathing dynamics, in which phases of pulse self-compression cyclically follow pulse-stretching phases (Fig. 4a,f). Strong high-order dispersion (HOD) decouples solitons with different *N* (Fig. 4a,f), breaking the cycles of soliton breathing^42^ and inducing soliton fission^49^. High-order dispersion, however, does not necessary arrest soliton self-compression. With a suitably tailored overall dispersion profile, HOD, as can be seen from Fig. 4b,g, does not prevent SSC, but, rather, makes it happen on a larger spatial scale. As the pulse width becomes close to the field cycle as a part of this dynamics, shock effects set in, making the trailing edge of the pulse steeper (Fig. 4h) and inducing a spectral blue shift (Figs. 4c, 5a).

At higher *W*_0_ and *p*, SSC tends to occur faster, that is, within a shorter propagation length. In this regime, shorter and more intense self-compressing soliton transients can be generated, leading to a stronger shock-induced pulse self-steepening and related blue shifting. At the level of *W*_0_ and *p* required for SSC to subcycle pulse widths, ionization effects set in (Figs. 4d,i, 5), causing a strong blue shift^50–55^. In the physical scenario considered in this study, where self-steepening couples to SSC to assist in the generation of subcycle pulses, ultrafast ionization is a major limiting mechanism, as it induces significant loss of radiation energy.

**Figure 6:**
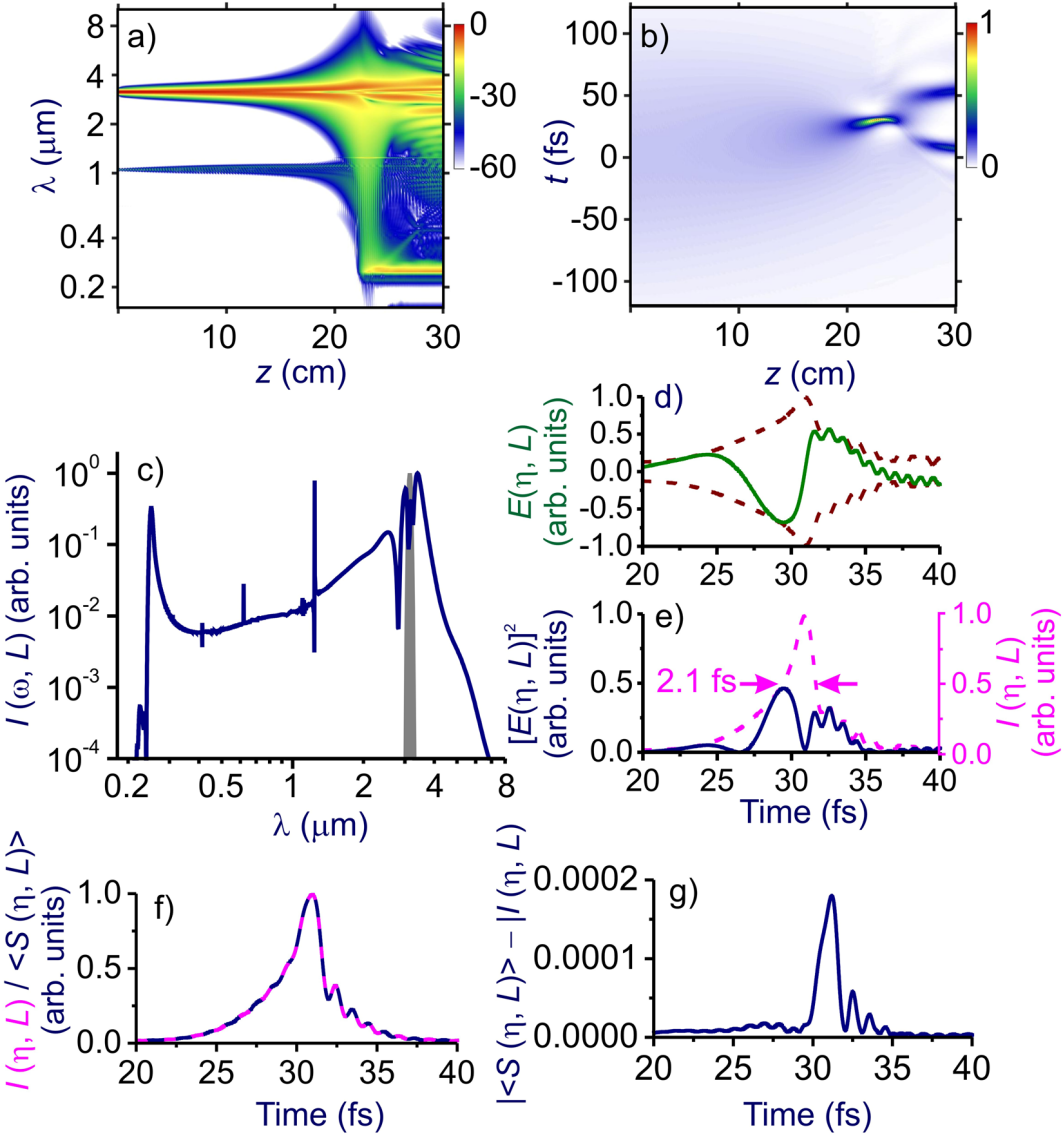
The maps of *I*(*ω, z*) (**a**) and *I*(*η, z*) (**b**) for a mid-IR driver in the single-ring hollow PCF. The fiber is filled with argon at *p* ≈ 16 bar. The input driver energy is *W*_0_ ≈ 18 μJ. (**c**) The spectral intensity *I*(*ω, L*), (**d**) the field *E*(*η, L*) (solid line) with its envelope (dashed line), and (**e**) [*E*(*η, L*)]^2^ (solid line) and intensity *I*(*η, L*) (dashed line) at the output of a hollow PCF filled with argon at *p* ≈ 16 bar. (**f**) The field intensity *I*(*η, L*) calculated using Eq. (6) (pink line) versus *<S*(*η, L*) > calculated using Eq. (5) (blue line). (**g**) Deviation | *<S*(*η, L*) > −*I*(*η, L*) | , with *S*(*η, L*) and *I*(*η, L*) calculated using Eq. (5) and (6), respectively. The input energy of the driver pulse is *W*_0_ ≈ 18 µJ. The fiber length is *L* = 23 cm. The input spectrum of the driver is shown by grey shadin.

Whether or not the shock-wave-induced enhancement of the spectral broadening of self-compressing solitons translates into shorter minimum pulse widths critically depends on the transmission bandwidth and the dispersion profile of the fiber. Simulations presented in Figs. 4 and 5 show that, with our choice of fiber design and parameters, a high-throughput SSC to pulse durations much shorter than the field cycle can be achieved. In Fig. 6, we present the spectrum of one of such pulses, produced via an SSC of input pulses with *W*_0_ ≈ 18 µJ in a ≈ 23-cm PCF with *p* ≈ 16 bar. At *z*_c_ = 23 cm, the SSC dynamics is seen to yield a field waveform featuring a central peak with full width at half-maximum (FWHM) as short as *τ*_c_ ≈ 2.1 fs (Fig. 6e), whose spectrum is centered at *λ*_c_ ≈ 2.1 μm and spans over several octaves (Fig. 6b,d,e). The FWHM pulse width of this peak is less than one-third of the field cycle at *λ*_c_ = 2.1 μm, with its energy estimated at 4.8 μJ (36% of the overall energy within the compressed pulse at *z*_c_ = 23.08 cm). The peak power of this soliton transient is ≈ 1.5 GW.

It is instructive to compare the field intensity *I*(*η, L*) calculated for such an extremely short field waveform using the approximation of Eq. (6) versus the rigorous definition of Eq. (4), with *S*(*η, L*) as given by Eq. (5). As can be seen from such a comparison, presented in Fig. 6f, results of approximate calculations of *I*(*η, L*) are indistinguishable (cf. pink and blue lines in Fig. 6f) from calculations performed using Eqs. (4) and (5) with *n*(*ω*) and *κ*(*ω*) profiles as shown in Fig. 1b,c. The deviation of *<S*(*η, L*) > calculated with the use of Eq. (5) from the field intensity *I*(*η, L*) as defined by the approximation of Eq. (6) is found to be within 0.02% (Fig. 6g).

## Conclusion

To summarize, we have identified regime of ultrafast nonlinear dynamics in which an optical shock wave couples to soliton self-compression, steepening the back of the pulse, yielding self-compressing soliton transients as short as the field sub-half-cycle. We have demonstrated that this extreme pulse self-compression scenario can help generate sub-half-cycle pulses in the mid-IR range in a broad class of anomalously dispersive optical waveguide systems, including specifically designed hollow-core fibers.

## Data Availability

All data generated or analyzed during this study are included in this published article.
